# History of toxicology at the Technical University of Munich (TUM)

**DOI:** 10.1007/s00210-024-03315-0

**Published:** 2024-07-24

**Authors:** Helmut Greim

**Affiliations:** Technical University of Munich, Munich, Germany

**Keywords:** Technical University of Munich, Clinical toxicology, Toxicology, Toxicology and Environmental Hygiene, Klinikum rechts der Isar, Helmholtz-Institute of Molecular Toxicology and Pharmacology, Biographies, History

## Abstract

Toxicology at the TUM is mainly associated with the Faculty of Medicine at the Klinikum rechts der Isar (MRI). The Department of Clinical Toxicology has been founded in 1963. Max von Clarmann, the head, focused his activities on the treatment of intoxications and the development of analytical methods and established a poison information center. His successors, Thomas Zielker and Florian Eyer, further developed this department to an internationally renown institution.

In 1967, the MRI became the TUM faculty of medicine with its Institute of Pharmacology and Toxicology. The director Melchior Reiter, formerly Institute of Pharmacology of the Ludwig Maximilians University (LMU), in 1970 initiated the foundation of the Department of Toxicology at the Gesellschaft für Strahlen- und Umweltforschung (GSF) with the director Gerhard Lange. The research focused on the neurotoxic effects of heavy metals and the metabolism and hepatoxicity of persistent chemicals. After Lange’s unexpected death in 1973, he was succeeded in 1975 by Helmut Greim from the University of Tübingen. The now Institute of Toxicology rapidly expanded developing and standardizing in vitro test methods, investigating the mechanism of carcinogens and mutagens and heavy metal toxicity. Training courses in the 15 major areas of toxicology have been organized at the GSF and competent centers in Germany. In 1987, Greim became the director of the newly founded Institute of Toxicology and Environmental Hygiene of the TUM, with expanded research and teaching activities, especially in toxicology at the faculties of Chemistry of the TUM and LMU, which thereafter became mandatory for students of chemistry at German universities.

## Introduction

Whereas toxicology in the Institute of Pharmacology of the Ludwig-Maximilian-University of Munich has a long-lasting tradition, toxicology at the TUM started with a Dept. of Clinical Toxicology in the Klinikum rechts der Isar (MRI), at that time, a hospital of the City of Munich. When in 1967 the TUM founded its Faculty of Medicine the MRI became the medical clinic of the new faculty. In addition, the necessary theoretical disciplines and among others the Institute of Pharmacology and Toxicology had to be founded. The elected chair of the new Institute of Pharmacology and Toxicology was Melchior Reiter, formerly a professor at the LMU Institute of Pharmacology. At the same time, Reiter chaired a small unit of pharmacology at the GSF, a federal research institute in a suburb of Munich. There, Reiter recommended to establish an Institute of Toxicology with the focus on the potential adverse health effects of environmental chemicals. The institute rapidly expanded and became one of the leading research institutes of toxicology in Germany with a great international reputation. More details about the foundation and activities of the different institutes involved in toxicology research and teaching are presented.

## The department of (clinical) toxicology

Toxicology at the TUM is mainly associated with the Faculty of Medicine at the Klinikum rechts der Isar (MRI). Before the clinic became part of the TUM, the clinician Max von Clarmann founded the department of (clinical) toxicology within the II. Clinic of Internal Medicine in 1963. His successors, Professors Thomas Zilker and Florian Eyer, further developed this department to an internationally renowned institution contributing a great number of publications on the identification and treatment of intoxications. Among them are intoxications by organophosphates, mushrooms, cyanide, and cyanide via smoke inhalation in fire, colchicine, drug abuse, lithium, alcohol, and their treatment and the development and evaluation of new treatment schedules (Eyer and Zilker 2010; Zilker and Eyer 2016; White et al. 2018; Popp et al. 2020; Whitfield et al. 2021).

In 2023, Zilker published a comprehensive book on the identification and treatment of intoxications (Zilker 2023).

## Max von Clarmann (1928–2006)

**Fig. 1:**
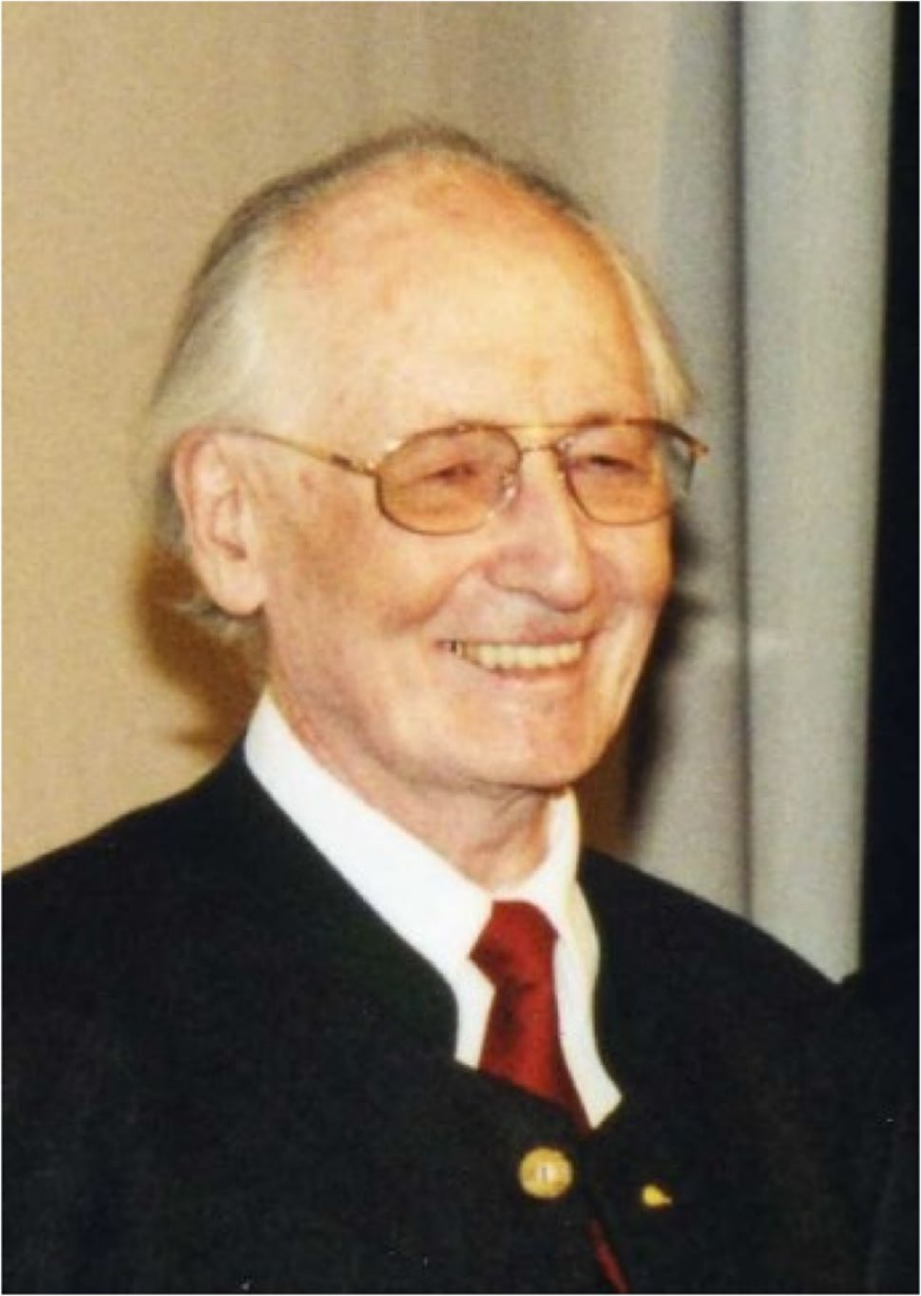
Max von Clarmann (GTFCh Homepage. T + K (2006) 73: 88. Personalia)

Max von Clarmann was born on April 29, 1928 (Fig. 1). After a 2-year nursing practice at the MRI, he studied medicine at the Ludwig Maximilians Universität (LMU) in Munich until 1952. Since then, he continuously has been affiliated with the MRI. Since 1955, he became interested in intoxications and their treatment, and his engagement over the years became acknowledged by the klinikum resulting in the foundation of a Dept. of Clinical Toxicology, headed by Max von Clarmann. Under his very effective guidance, the department grew rapidly focusing on the treatment of intoxications, the development of analytical methods, and finally establishment of a poison information center. The acquirement of simple analytical methods has been considered of great importance to allow a rapid identification of the poison, which he conceded crucial for the necessary therapeutic measures. Max von Clarmann died in Munich on June 20, 2006.

## The institutes of toxicology and of toxicology and environmental hygiene

In 1967, the MRI became the TUM faculty of medicine with the Institute of Pharmacology and Toxicology founded in 1968. Prof. Melchior Reiter, the director, formerly Institute of Pharmacology of the Ludwig Maximilians University (LMU), has been interested in mechanisms of glycosides with some activities on heavy metal toxicity, especially the metabolism of mercury and its organic compounds. He initiated the foundation of the Institute of Toxicology at the Gesellschaft für Strahlen- und Umweltforschung (GSF).

## Melchior Reiter (1919–2007)

**Fig. 2:**
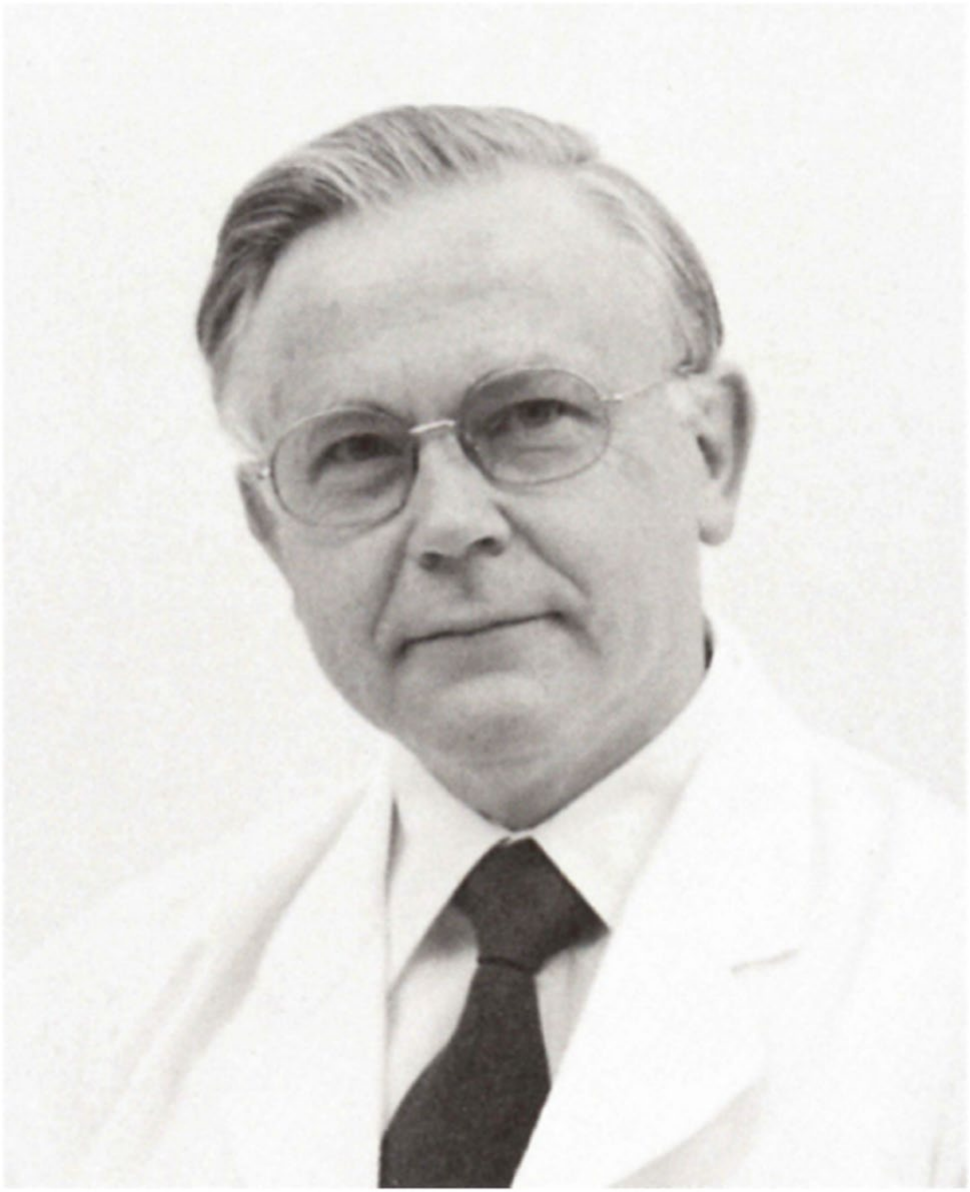
Melchior Reiter (archive of DGPT)

Melchior Reiter, born in Berlin 1919, studied medicine in Berlin, and in 1944 received his M.D. at the Berlin Institute of Pharmacology of Prof. W. Heubner (Fig. 2). Shortly after WW II, he received a grant to join Otto Krayer’s Institute of Pharmacology at the Boston Medical School. Krayer left Germany in 1933 after he had declined the departmental chair position at the University of Düsseldorf of Philipp Ellinger, who had been removed from the chair on racial grounds. Back in Germany, Reiter received the position of an assistant of the LMU Institute of Pharmacology in Munich, starting his research on the mechanism of amanitin intoxication, published in the Festschrift of W. Heubner’s 75th birthday together with Otto Wieland and Hans Georg Fischer (Wieland et al 1952). Between 1970 and 1987, he also chaired the Dept. of Pharmacology of the GSF near Munich. From 1979 to 1981, he was Dean of the TUM Faculty of Medicine. In the TUM Institute research focused on cyclic AMP (Honerjäger and Schäfer-Korting 1981), relaxant effects on smooth muscles (Reiter and Brandt (1985), or cardiac calcium channels (Reiter 1988).

Reiter once reported an interesting event during the year before WW II. In 1938, he served as a mountain infantryman of the German army in Berchtesgaden near the Austrian border. When the German army has been ordered to enter Austria, his unit marching from Berchtesgaden uphill to the Austrian border was not so sure whether they will be welcome. Arriving at the border, it was their great relief that the border was open and no Austrian Military around. They happily marched downhill to the Austrian city of Hallein, where they have been welcomed by a music band.

In 1970, Reiter, together with Prof. Friedhelm Korte, Director of the Institute of Ecological Chemistry at the GSF and the TUM in Weihenstephan, proposed the foundation of a working group of Toxicology at the GSF. Prof. Gerhard Lange, also from the LMU Institute of Pharmacology, has been appointed as director. Research focused on neurotoxicity and aquatic toxicity, toxicity of heavy metals, and organic pollutants, as well as metabolism and hepatoxicity of persistent chemicals such as polychlorinated biphenyls. After the unexpected death of Gerhard Lange in 1973, the development of the group stagnated until Helmut Greim from the University of Tübingen has been appointed as director in 1975. Melchior Reiter died on February 18, 2007, in Prien/Bavaria, a small city in the Alps.

## Helmut Greim (1935)

Helmut Greim, born May 9, 1935, in Berlin, studied medicine at the University of Freiburg and the Free University of Berlin. Based on his experimental work on the digestion of infants with diarrhea, he received his MD in 1962 and thereafter joined the research group of Prof. Herbert Remmer at the Institute of Pharmacology (director Prof. Hans Herken) of the Free University. When Remmer in 1965 became Director of the Institute of Toxicology, University of Tübingen, Greim followed him receiving the position of an assistant at the institute. There he continued his research on cytochrome P450, and in 1970, after determining the half-lives of at that time only known two P450 enzymes received his habilitation. Thereafter, he accepted the position of a visiting Research Associate Professor at the Dept. of Pathology, Mount Sinai School of Medicine of the City University of New York (Chair and Dean Dr. Hans Popper). During this time, he also held the position of Visiting Fellow of Pharmacology at the Yale School of Medicine in New Haven, Connecticut. Research at Mount Sinai focused on the P450-mediated metabolism of bile salts during cholestasis (Greim et al. 1972), of carcinogens and mutagens and the introduction of Phase I metabolic activity into the Ames test (Popper et al. 1973). These research activities continued in Tübingen after Greim’s return to the institute in 1973.

**Fig. 3:**
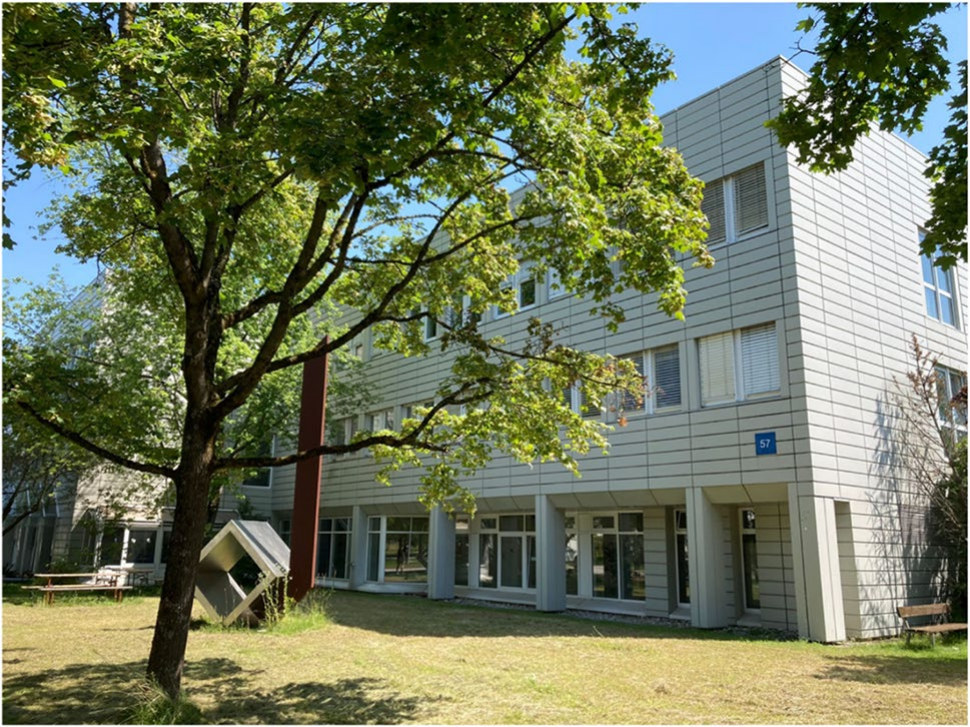
The “Tox Building” of the GSF in Neuherberg (provided by Prof. Hans Zischka)

After Greim became director of the GSF toxicology in 1975, the group expanded rapidly and became an institute in 1978. In 1988, the institute moved into the new and spacy well-equipped building (The “Tox Building”) (Fig. 3).

Research focused on developing and standardizing in vitro test methods including characterization of metabolizing enzymes of commonly in toxicology used cell lines, establishing rat hepatocytes and V79 cells with competent metabolizing capacity to investigate cytotoxicity, genotoxicity, and DNA repair of chemicals (Greim et al. 1975, 1980; Doehmer et al. 1994).

Studies on the mechanism of carcinogenesis and mutagenesis included intercellular communication, cell proliferation, and apoptosis, and to extrapolate results from animal studies to humans, physiological-based pharmacokinetic (PBPK) models have been developed and applied for many carcinogens (Peter et al. 1987). The toxicology of metals included molecular mechanisms of copper exposure of infants and biomonitoring of mercury after the removal of amalgam fillings.

Participation in the “reactive oxygen club” organized by Prof. E. Elstner, Institute of Biology und Microbiology of the TUM, improved the understanding of consequences of inflammation resulting in the reactive oxygen species during inflammation and cytotoxicity.

Together with the toxicologist Frederick Coulston, Albany Medical College, New York State, USA, the institute became responsible for the research activities at the former NASA facility in Alamogordo, New Mexico, which kept monkeys and apes for research purposes. From the German side, this has been supported by the Federal Ministry of Research and Development. There, several studies on the effects and elimination of dioxins in rhesus monkeys have been conducted (Rozman et al. 1981). After the end of these activities, several members of the GSF Institute of Toxicology stayed in the USA and received positions at the Institute of Toxicology, Kansas City University. One of them, Dr. Karl Rozman, became a member of the US TLV (threshold limit values) committee of the American Conference of Governmental Industrial Hygienists (ACGIH) and served as a liaison officer between the German MAK Commission and the TLV committee.

**Fig. 4:**
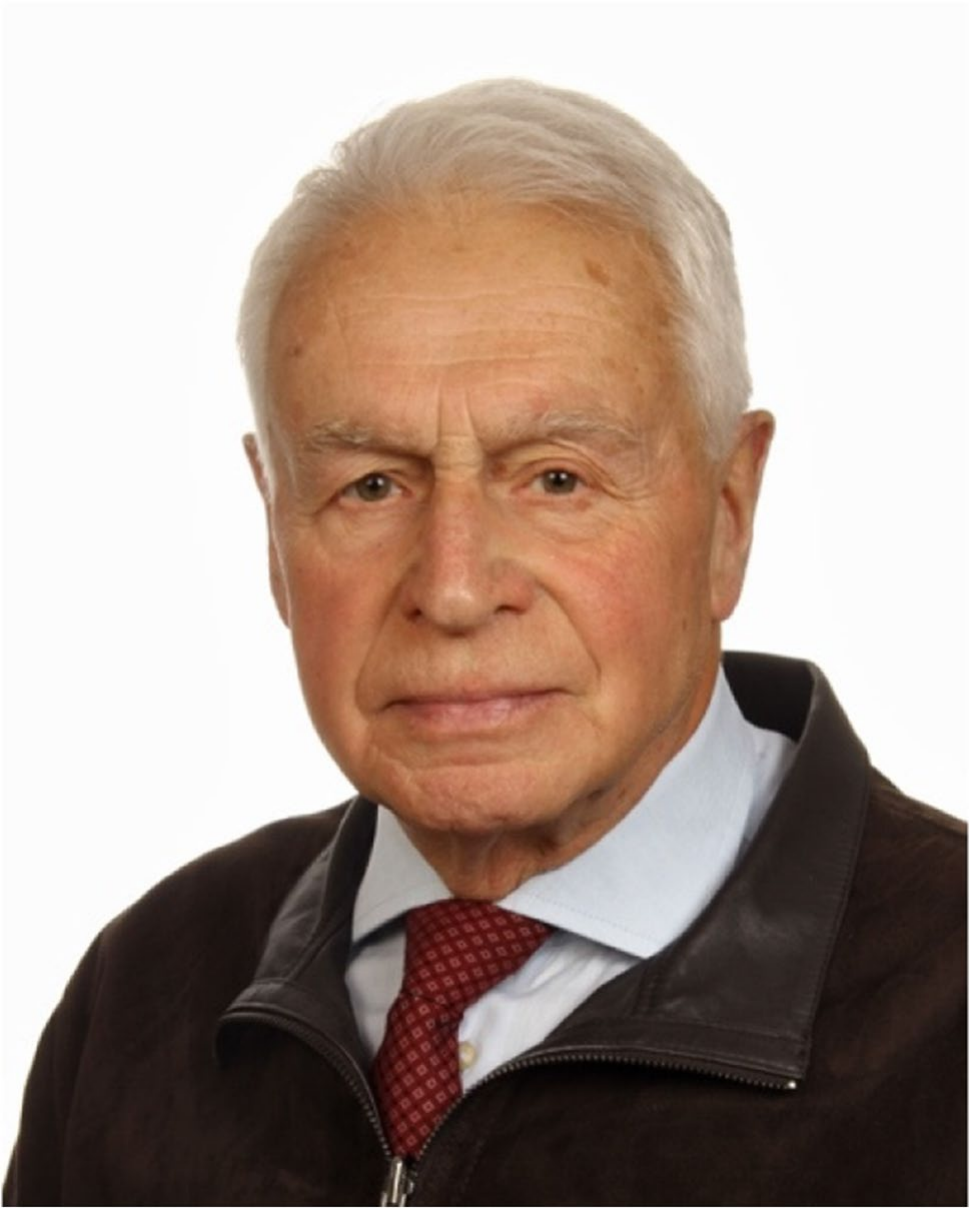
Helmut Greim (private)

In 1987, after declining the positions of chair and director of toxicology at the Universities of Tübingen and Vienna, Austria, Greim became director of the newly founded Institute of Toxicology and Environmental Hygiene of the TUM (Fig. 4). This further expanded research and teaching activities in toxicology for medical students but also for students of chemistry at both Munich universities. With the assistance of the president of the TUM, Prof. Wolfgang Hermann, and the vice president of the University of Tübingen, Prof. Ernst Bayer, both chemists, lectures in toxicology for students in chemistry became mandatory for students of chemistry at German universities.

**Fig. 5:**
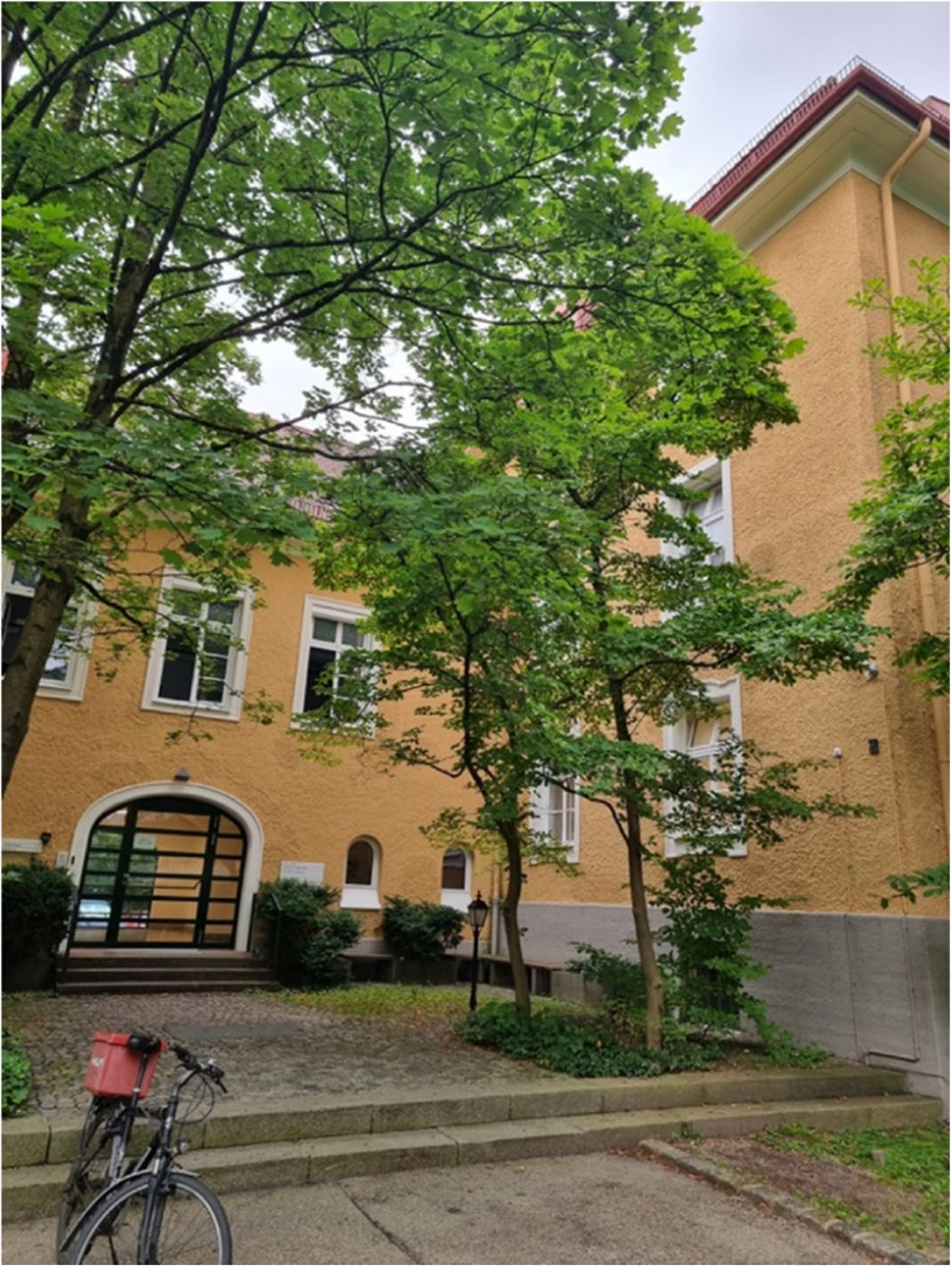
Institute of Toxicology and Environmental Hygiene of the TUM (provided by Prof. Hans Zischka)

At the time when Greim retired in 2000, the institutes had a total of 120 coworkers including the scientific secretariate of the MAK-Committee (Commission for the Investigation of Health Hazards of Chemicals in the Work Area of the German Research Foundation), which Greim chaired. In 2003, his successor, Prof. Dr. Martin Göttlicher, became director of both the TUM Institute of Toxicology and Environmental Hygiene (Fig. 5) and the GSF Institute of Toxicology, now the Helmholtz-Institute of Molecular Toxicology and Pharmacology, specifically investigating the molecular mechanisms of bioactive chemicals with the specific aim to develop effective and safe drugs (Einer et al. 2023).

In the institutes of Food Chemistry and Analytical Food Chemistry of the TUM School of Life Sciences in Weihenstephan, phytotoxins are identified and evaluated regarding their relevance for food safety.

## Training courses in toxicology

Starting in 1975, a postgraduate training program in toxicology has been established at the GSF institute, which since 1980 has comprised courses of the 15 major areas of toxicology. These courses are organized on behalf of the German Society of Toxicology and are held in the Munich institutes and competent centers in Germany. They provide the knowledge necessary to finally receive the title “Certified Toxicologist” by the German Society of Toxicology. This title also meets specific requirements in education as well as professional skills and experience for the title “European Registered Toxicologist” (ERT). At present, more than 600 postgraduate students are enrolled to attend the now 17 different courses, which presently are organized by the TUM institute.

## Participation in scientific advisory committees

Due to their broad competence in toxicology and risk assessment, members of the institute chaired or participated in scientific advisory committees. These include the “MAK Committee,” the Advisory Committee on Existing Chemicals (BUA), a committee of the Society of German Chemists (GDCH), of both, the scientific secretariates have been in the GSF and TUM institutes. Participation in other committees included the Scientific Committee of Health and Environmental Risks (SCHER) and the Scientific Committee of Occupational Exposure Limits (SCOEL) of the European Commission, and the Risk Assessment Committee (RAC) of the European Chemicals Agency (ECHA). In addition, Greim has been a member of the Enquète Commission “Men and the Environment” of the German Parliament and the German Advisory Council on the Environment (Rat der Sachverständigen für Umweltfragen) of the Federal Ministry for the Environment, Nature Conservation, Nuclear Safety and Consumer Protection.

## Risk assessment of specific compounds

The participation in scientific advisory committees of members of the institute resulted in a broad experience in the risk assessment of chemicals and other materials. The results of these assessments have been published in the open literature. Among these are publications on the toxicology of particles (Greim and Ziegler-Skylakakis 1997), refractory ceramic fibers (Greim et al. 2014a), the herbicide glyphosate (Williams et al. 2016), the relevance of synthetic endocrine disruptors (Autrup et al. 2020), or emissions from diesel engines (Greim 2019).

## Organization of international meetings

1988: annual meeting of the European Society of Toxicology (EUROTOX), about 150 participants from Europe and the USA.

1998: the XIII International Congress of Pharmacology with Helmut Greim as President together with Prof. Franz Hofmann after he became Director of the TUM Institute of Pharmacology and Toxicology. Over 6000 participants.

2004: Recent Advances in Benzene Toxicity III, about 150 participants.

2009: Recent Advances in Benzene Toxicity IV, about 150 participants.

2014: The last “Benzene Meeting” at the New York Academy of Sciences: The bone marrow niche, stem cells, and leukemia: impact of drugs, chemicals, and the environment, about 150 participants (Greim et al. 2014b).

## Data Availability

No datasets were generated or analysed during the current study.
